# Red light exaggerated sepsis-induced learning impairments and anxiety-like behaviors

**DOI:** 10.18632/aging.103940

**Published:** 2020-11-10

**Authors:** Bing Xie, Yujing Zhang, Hong Qi, Hua Yao, You Shang, Shiying Yuan, Jiancheng Zhang

**Affiliations:** 1 Department of Critical Care Medicine, Union Hospital, Tongji Medical College, Huazhong University of Science and Technology, Wuhan 430022, China; 2 Institute of Anesthesia and Critical Care Medicine, Union Hospital, Tongji Medical College, Huazhong University of Science and Technology, Wuhan 430022, China

**Keywords:** light exposure, gut microbiota, sepsis-associated encephalopathy, spleen, subdiaphragmatic vagus nerve

## Abstract

Light exerts critical non-visual effects on a multitude of physiological processes and behaviors, including sleep-wake behavior and cognitive function. In this study, we investigated the effects of continued exposure to different colors of light on cognitive function after sepsis in old mice. We found that exposure to red light, but not green light, exaggerated learning impairments and anxiety-like behaviors after sepsis. Red light also induced remarkable splenomegaly and altered the diversity and composition of the fecal microbiota. Pseudo germ-free mice transplanted with fecal bacteria from septic mice exposed to red light developed the same behavioral defects and splenomegaly as their donors. Intriguingly, splenectomy and subdiaphragmatic vagotomy reversed the learning impairments and anxiety-like behaviors resulting from red light exposure after sepsis. After subdiaphragmatic vagotomy, no differences in behavior or spleen size were observed among pseudo germ-free mice transplanted with fecal bacteria from septic mice exposed to different colors of light. Our results suggested that red light exposure after sepsis in old mice causes gut microbiota dysfunction, thus stimulating signaling through the subdiaphragmatic vagus nerve that induces splenomegaly and aggravates learning impairments and anxiety-like behaviors.

## INTRODUCTION

Sepsis is systemic inflammation in response to a bacterial infection. Sepsis is frequently complicated by sepsis-associated encephalopathy (SAE), which can range from mild delirium to a deep coma [1]. Even in the absence of an overt central nervous system infection, SAE can cause long-term cognitive impairment, anxiety and stress disorders [2], placing a great burden on families and social systems. The etiology of SAE is not fully understood, but excessive inflammatory responses [3], oxidative stress and blood-brain barrier disruption [4, 5] have been hypothesized to contribute to its pathophysiology. In view of the high mortality rate and poor prognosis of patients with SAE [6], effective therapy is warranted.

The human intestinal tract harbors a diverse community of bacterial species, collectively called the gut microbiota [7]. The gut microbiota are widely accepted to connect the gut and the brain in what is known as the microbiota-gut-brain axis [8]. The structure of the gut microbial community can be altered markedly by endogenous and exogenous factors such as antibiotics, probiotics, dietary components and infections [9–12]. Imbalances in the gut microbiota can increase the permeability of the gut and the blood-brain barrier, facilitating the inflammatory processes of diseases associated with neuroinflammation [13]. Abnormal gut microbiota have been linked to the pathogenesis of neurobehavioral disorders such as anxiety and depression [14, 15]. The gut microbiota are known to impact the host’s immune response and survival following sepsis [16]; however, the involvement of the gut microbiota in the development of SAE remains largely unknown. Although the pathways whereby the gut microbiota communicate with the brain are controversial, the vagus nerve has been reported to participate in bacteria-brain signaling [17–19].

The mammalian retina not only distinguishes colors, but also contributes to physiological and behavioral functions such as sleep-wake regulation, hormone secretion, heart rate regulation and body temperature modulation [20–23]. These functions depend on a subset of intrinsically photosensitive retinal ganglion cells (ipRGCs) that express the photopigment melanopsin in the retina [20, 24]. Different subpopulations of ipRGCs project to various regions of the brain, where they trigger diverse physiological responses. For example, alertness is associated with suprachiasmatic nuclei, while sleep propensity depends on projections to the ventrolateral preoptic area [20, 21]. There is also evidence that ipRGCs project to memory- and emotion-related areas such as the hippocampus and amygdala [25–27], indicating that light actively regulates cognitive processes. Green wavelengths have been reported to promote sleep [21, 28].

In this study, we investigated the effects of exposure to different colors of light on cognitive function after lipopolysaccharide (LPS)-induced sepsis in old mice. We also examined the effects of colored light exposure on the composition of the gut microbiota and the size of the spleen after sepsis. By transplanting fecal bacteria from red light-exposed septic mice into pseudo germ-free mice, we explored the contribution of the gut microbiome to the pathophysiological processes of cognitive disorders. Finally, we assessed the involvement of the spleen and the subdiaphragmatic vagus nerve in these behavioral deficits by performing subdiaphragmatic vagotomy (SDV) and splenectomy.

## RESULTS

### Red light exposure impaired cognitive and neuropsychiatric function in non-septic and septic mice

**Figure 1:**
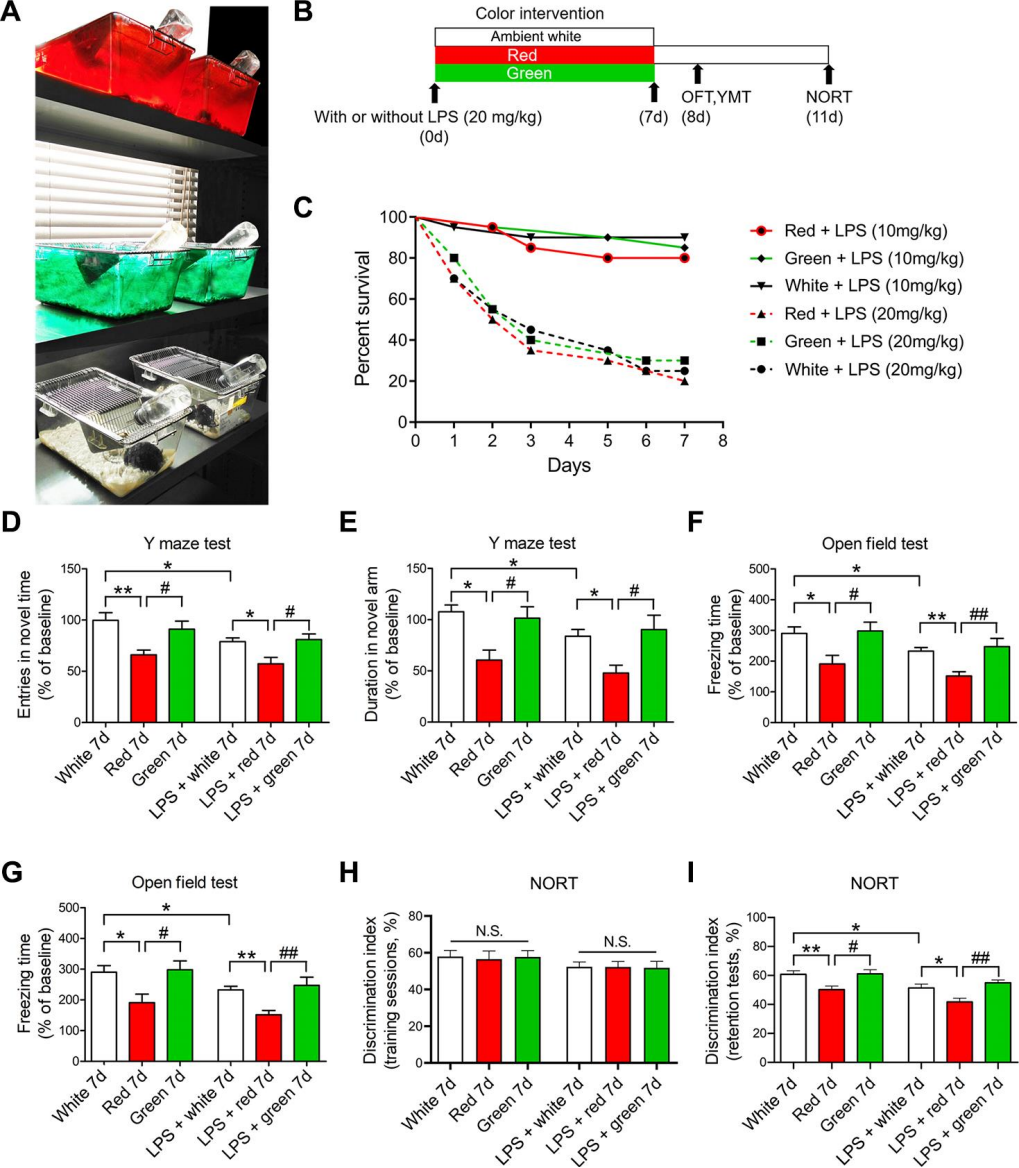
**Red light exposure induces cognitive impairment in non-LPS-treated and LPS-treated mice.** (**A**, **B**) Treatment schedule. Mice were treated with or without LPS (20 mg/kg), and then were exposed to light for seven days. Non-LPS-treated mice and LPS-treated mice were scheduled for open field and Y maze tests on day 8 and a NORT on day 11. (**C**) Survival curves (n = 20/group). The survival rate did not differ significantly among the red, green and ambient white light-exposed groups seven days after the administration of LPS (10 or 20 mg/kg). (**D**–**I**) Red light exposure impaired spatial learning and memory and induced anxiety-like behavior compared with ambient white light and green light exposure in mice, as evidenced by the reduced frequency of entering the novel arm (**D**) and the reduced time spent in the novel arm (**E**) in the Y maze test, the reduced time spent in the center (**F**) and the reduced freezing time (**G**) in the open field test, and the reduced time exploring the novel object (**H**, **I**) in the NORT. Data are shown as the mean ± SEM (n = 6-8/group). ^*^P < 0.05, ^**^P < 0.01, ^#^*P* < 0.05, ^##^*P* < 0.01.

To investigate the effects of red and green light on cognitive and neuropsychiatric function in septic mice, we induced sepsis using LPS and exposed the mice to different colors of light for seven days. Open field and Y maze tests were conducted on day 8, and a novel object recognition task (NORT) was performed on day 11 (Figure 1A, 1B). The survival rate did not differ significantly among mice exposed to red, green or ambient white for seven days after treatment with different doses of LPS (10 or 20 mg/kg, Figure 1C; *P* > 0.05). LPS administration at a dose of 20 mg/kg induced SAE, as demonstrated by a lower frequency of entering the novel arm and a shorter time spent in the novel arm in the Y maze test (Figure 1D, 1E; both *P* < 0.05), a shorter time exploring the center of the open field and a shorter freezing time in the open field test (Figure 1F, 1G; both *P* < 0.05), and a shorter time exploring the novel object in the NORT (Figure 1I; *P* < 0.05) in LPS-treated mice than in mice treated with 0.9% saline.

Interestingly, in the Y maze test, both the frequency of entering the novel arm and the time spent in the novel arm were significantly lower in 20 mg/kg LPS-induced septic mice exposed to red light than in those exposed to ambient white light or green light (Figure 1D, 1E; all *P* < 0.05). Similarly, in the open field test, the time exploring the center of the open field and the freezing time were both shorter in the red light-exposed septic mice than in the other two groups (Figure 1F, 1G; all *P* < 0.05). In the NORT, the mice in each group spent a comparable duration exploring each object during the training session (Figure 1H; *P* > 0.05). However, during the retention test performed one hour after the training, the septic mice exposed to red light spent significantly less time exploring the novel object than the other two groups (Figure 1I; *P* < 0.05 and *P* < 0.01, respectively). These data suggested that red light exposure impaired recognition memory and elicited anxiety-like behavior in septic mice.

Intriguingly, even without LPS administration, mice exposed to red light for seven days developed cognitive dysfunction, manifested as an impaired learning capability and increased anxious behavior (Figure 1D–1I). These results indicated that red light exposure evoked cognitive impairment in both septic and non-septic mice.

### Splenectomy reversed red light exposure-induced cognitive and neuropsychiatric dysfunction following sepsis

**Figure 2:**
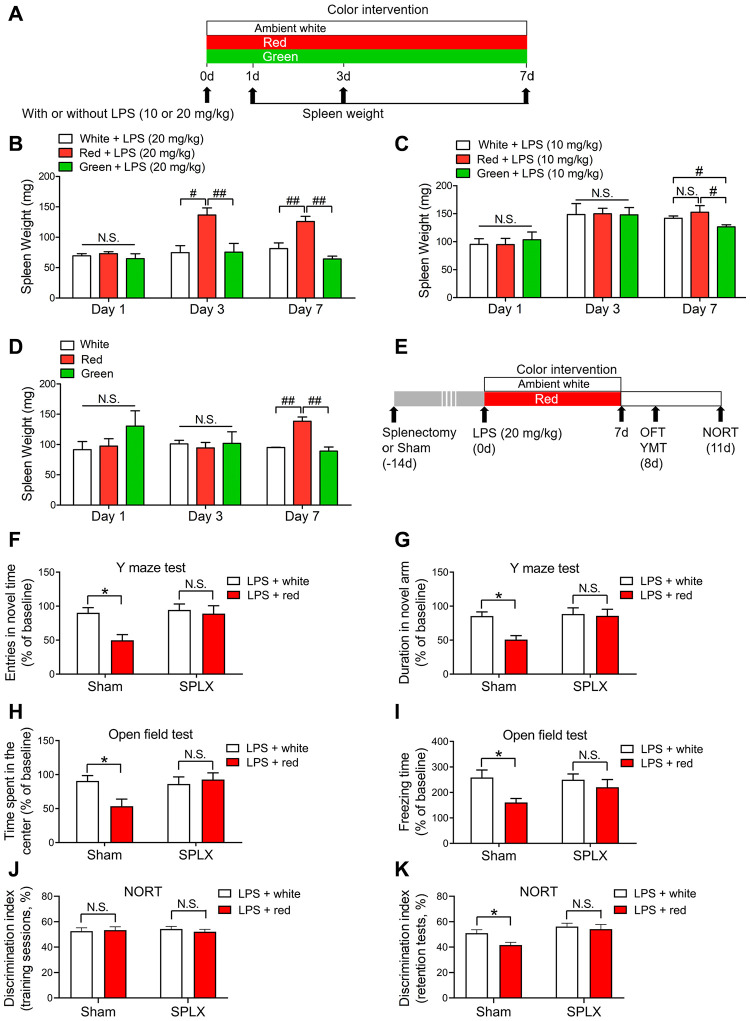
**Red light exposure elicits splenomegaly, while splenectomy reverses red light exposure-induced cognitive deficits in septic mice.** (**A**) Treatment schedule. Mice were treated with or without LPS (10 or 20 mg/kg), and then were exposed to light for up to seven days. The mice were euthanized and their spleens were collected on day 1, 3 or 7. (**B**) Red light exposure resulted in splenomegaly in LPS (20 mg/kg)-administered mice on days 3 and 7 relative to ambient white light or green light exposure. (**C**) Red light exposure led to splenomegaly in LPS (10 mg/kg)-treated mice on day 7 compared to green light exposure. (**D**) Red light exposure induced significant spleen enlargement in non-LPS-treated mice on day 7 relative to ambient white light and or green light exposure. (**E**) Treatment schedule. Mice underwent a splenectomy or sham surgery 14 days prior to LPS (20 mg/kg) administration, and were then exposed to light for seven days. On day 8, open field and Y maze tests were performed. On day 11, a NORT was performed. (**F**–**K**) Splenectomy reversed red light exposure-induced cognitive dysfunction and anxiety-like behavior, as demonstrated by the lack of significant difference in the frequency of entering the novel arm (**F**) and the time spent in the novel arm (**G**) in the Y maze test, the time spent in the center (**H**) and the freezing time (**I**) in the open field test, and the time exploring the novel object (**J**, **K**) in the NORT between splenectomized mice exposed to ambient white light or red light. Data are shown as the mean ± SEM (n = 6-8/group). N.S., not significant, ^*^P < 0.05, ^#^*P* < 0.05, ^##^*P* < 0.01.

While investigating the mechanisms underlying the red light exposure-induced brain disorders in mice, we unexpectedly observed remarkable splenomegaly in the red light-exposed group compared with the green light- and ambient white light-exposed groups on day 3 (*P* < 0.05 and *P* < 0.01, respectively) and day 7 (both *P* < 0.01) after the administration of 20 mg/kg LPS (Figure 2B). When mice were treated with 10 mg/kg LPS or simply exposed to different colors of light without LPS administration, no significant differences in spleen weight were observed among the three light-exposed groups on days 1 and 3, but a significant difference was found between the red and green light-exposed groups on day 7 (Figure 2C, 2D; P < 0.05 and P < 0.01, respectively). These results suggested that splenomegaly is associated with brain disorders.

In previous studies, the spleen was found to serve as a reservoir of inflammatory CD11b+ Ly-6Chigh monocytes in sepsis survivors [29, 30], and splenectomy prevented monocyte trafficking to the brain and attenuated anxiety-like behavior following sub-threshold stress in stress-sensitized mice [29]. Thus, we assessed whether splenectomy could protect against brain impairment following red light exposure (Figure 2E). We found that splenectomy abrogated the reduced frequency of entering the novel arm and the reduced time spent in the novel arm in the Y maze test (Figure 2F, 2G), the reduced time exploring the center of the open field and the reduced freezing time in the open field test (Figure 2H, 2I), and the reduced time exploring the novel object in the NORT (Figure 2J, 2K) in red light-exposed mice treated with 20 mg/kg LPS. These results indicated that the spleen was an essential contributor to red light exposure-induced memory disorders and anxiety-like behaviors.

### The effects of exposure to different colors of light on the fecal microbial diversity of non-septic and septic mice

**Figure 3:**
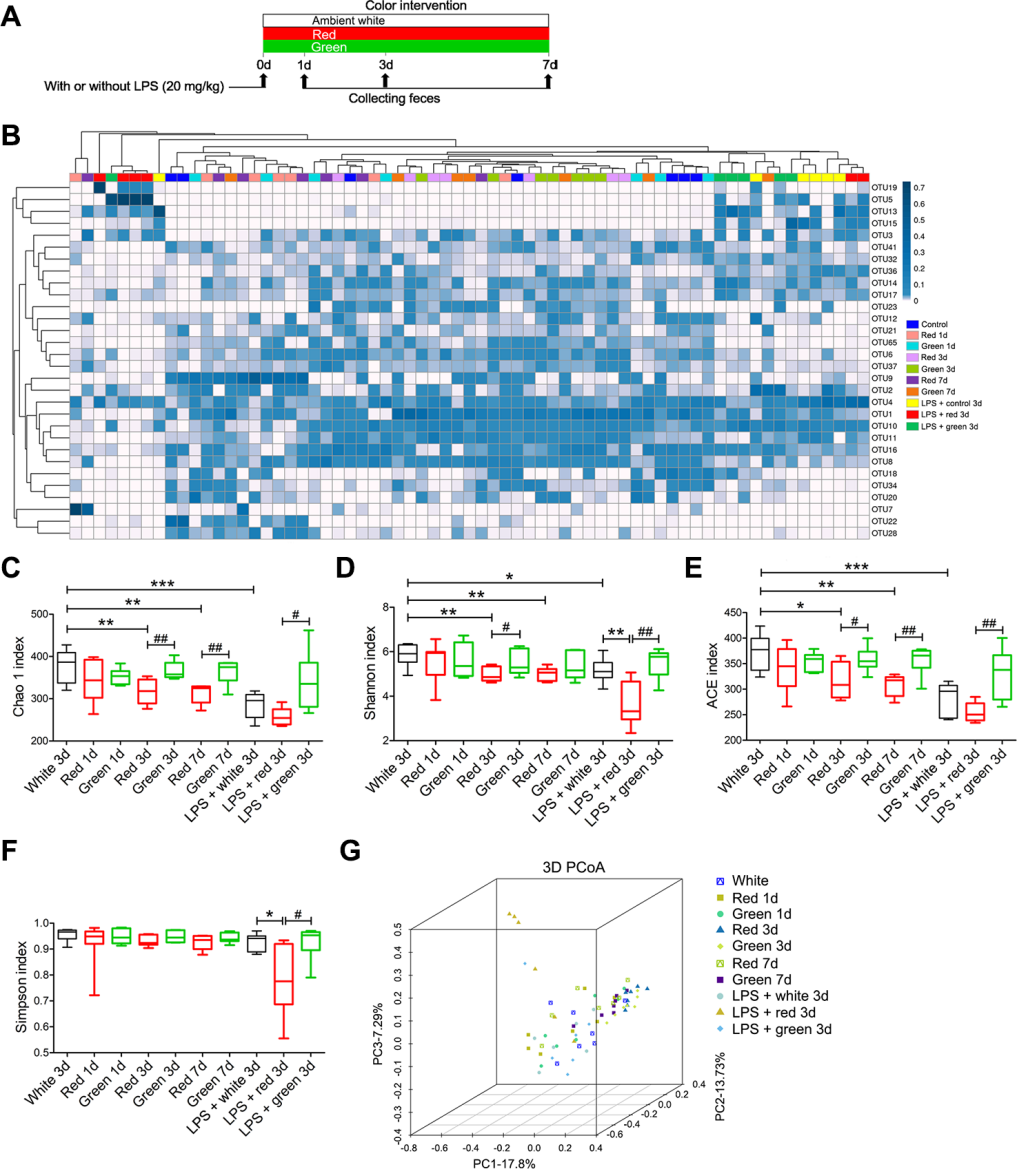
**Differential gut microbial profiles of non-septic and septic mice after light exposure.** (**A**) Treatment schedule. Mice were treated with or without LPS (20 mg/kg), and then were exposed to light for seven days. Fresh feces were collected on days 1, 3 and 7 for 16S rRNA gene sequencing. (**B**) Heat map of the fecal bacterial abundances in the different groups. LPS administration and red light exposure reduced the richness of the intestinal flora and altered the gut microbial composition, as determined using the Chao 1 index (**C**), Shannon index (**D**), ACE index (**E**), Simpson index (**F**) and 3D principal coordinate analysis (**G**). Data are presented as the mean ± SEM (n = 6-7/group). ^*^P < 0.05, ^**^P < 0.01, ^***^P < 0.001, ^#^*P* < 0.05, ^##^*P* < 0.01.

Given the previously reported effects of the gut microbiota on sepsis and cognition, we sought to determine whether red light exposure promoted cognitive dysfunction by altering the diversity and composition of the gut microbiota. Alpha diversity is a measure of the richness of the gut microbiota, and can be evaluated by the Chao 1, Shannon, ACE and Simpson indexes. We found that the Chao 1, Shannon and ACE indexes were significantly lower in fecal samples from septic mice than in those from non-septic mice (Figure 3B–3E; *P* < 0.001, *P* < 0.05 and *P* < 0.001, respectively). In non-septic mice, the Chao 1, Shannon and ACE indexes were significantly lower in the red light-exposed group than in the ambient white light-exposed group on day 3 (*P* < 0.01, *P* < 0.01 and *P* < 0.05, respectively) and day 7 (all *P* < 0.01) (Figure 3C–3E). The Chao 1 and ACE indexes in non-septic mice were significantly lower in the red light-exposed

group than in the green light-exposed group on day 3 (*P* < 0.01 and *P* < 0.05, respectively) and day 7 (both *P* < 0.01), whereas the Shannon index was significantly lower in the red light-exposed group than in the green light-exposed group only on day 3 (*P* < 0.05) (Figure 3C–3E). In septic mice, the Shannon and Simpson indexes were significantly lower in the red light-exposed group than in the ambient white light-exposed group on day 3 (Figure 3D, 3F; *P* < 0.01 and *P* < 0.05, respectively). In addition, the Chao 1, Shannon, ACE and Simpson indexes in septic mice were significantly lower in the red light-exposed group than in the green light-exposed group on day 3 (Figure 3C–3F; *P* < 0.05, *P* < 0.01, *P* < 0.01 and *P* < 0.05, respectively). These data suggested that red light exposure greatly reduced the fecal microbial diversity in mice with or without LPS treatment, while green light exposure had no effect on the fecal microbiota after sepsis. Accordingly, in a 3D principal coordinate analysis, the dots representing the red light-exposed septic group were far from the dots representing the ambient white light- and green light-exposed septic groups (Figure 3G).

### Alterations in the gut microbial composition in mice exposed to different colors of light

**Figure 4:**
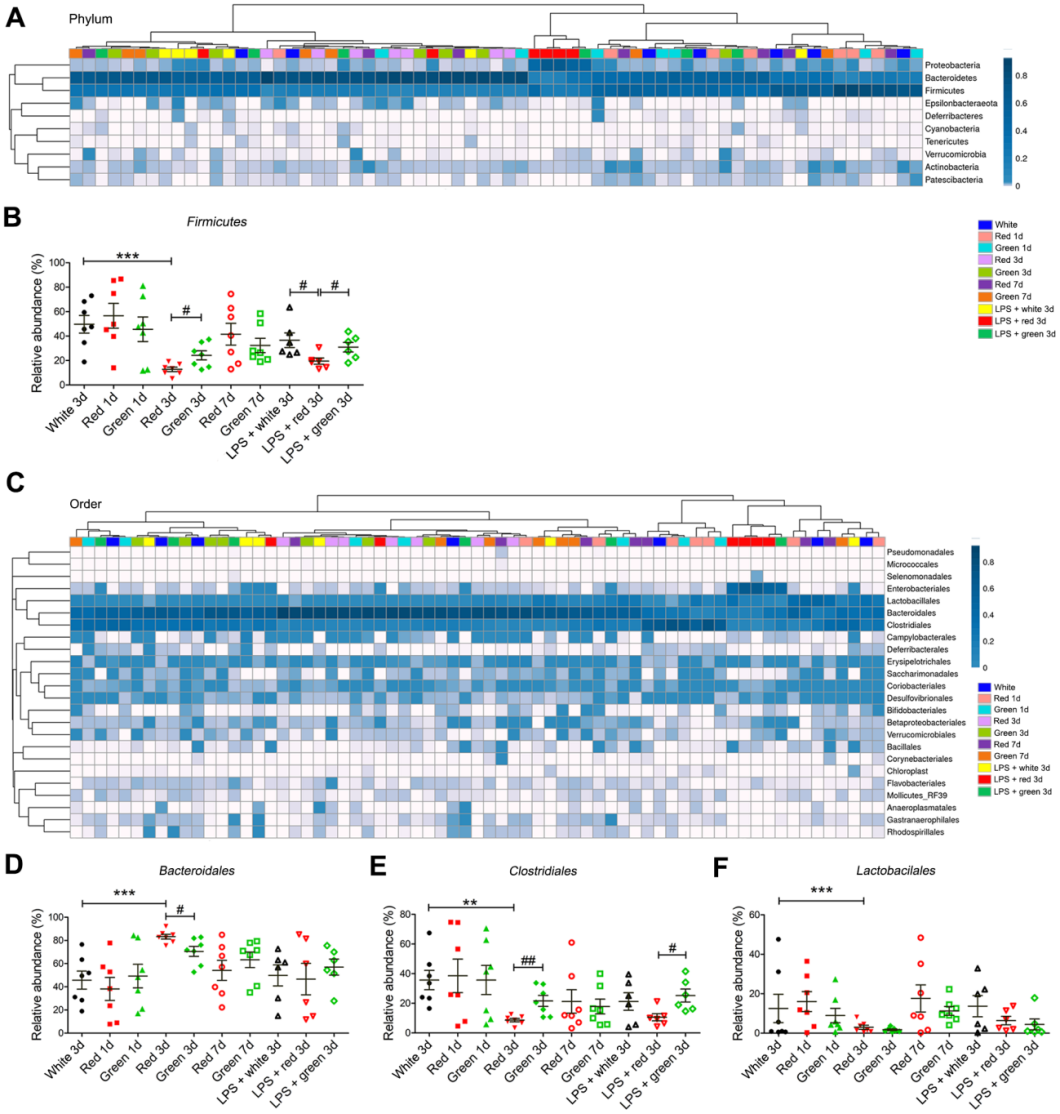
**Altered gut bacterial composition at the phylum and order levels.** (**A**) Relative abundance at the phylum level in the different groups. (**B**) Red light exposure reduced the abundance of *Firmicutes* in both non-septic and septic mice on day 3 relative to ambient white light or green light exposure. (**C**) Relative abundance at the order level in the different groups. (**D**) Red light exposure increased the abundance of *Bacteroidales* in non-LPS-treated mice on day 3 relative to ambient white light or green light exposure. (**E**) Red light exposure reduced the abundance of *Clostridiales* in non-septic mice on day 3 relative to ambient white light or green light exposure, as well as in septic mice on day 3 relative to green light exposure. (**F**) Red light exposure reduced the abundance of *Lactobacillales* in non-LPS-treated mice on day 3 relative to ambient white light exposure. Data are presented as the mean ± SEM (n = 6-7/group). ^**^P < 0.01, ^***^P < 0.001, ^#^*P* < 0.05, ^##^*P* < 0.01.

Next, we analyzed the gut microbial compositions of the mice in the different groups. At the phylum level, in non-septic mice, the relative abundance of *Firmicutes* was significantly lower in the red light-exposed group than in the ambient white light- and green light-exposed groups on day 3 (Figure 4A, 4B; *P* < 0.001 and *P* < 0.05, respectively).

At the order level, in non-septic mice on day 3, the red light-exposed group had a significantly greater relative abundance of *Bacteroidales* (*P* < 0.001 and *P* < 0.05, respectively) and a significantly lower abundance of *Clostridiales* (both *P* < 0.01) than the ambient white light- and green light-exposed groups (Figure 4C–4E). In addition, the relative abundance of *Lactobacillales* on day 3 in non-septic mice was significantly lower in the red light-exposed group than in the ambient white light-exposed group (Figure 4F; *P* < 0.001). In LPS-treated mice, the relative abundance of *Clostridiales* was significantly lower in the red light-exposed group than in the green light-exposed group (Figure 4E; *P* < 0.05).

**Figure 5:**
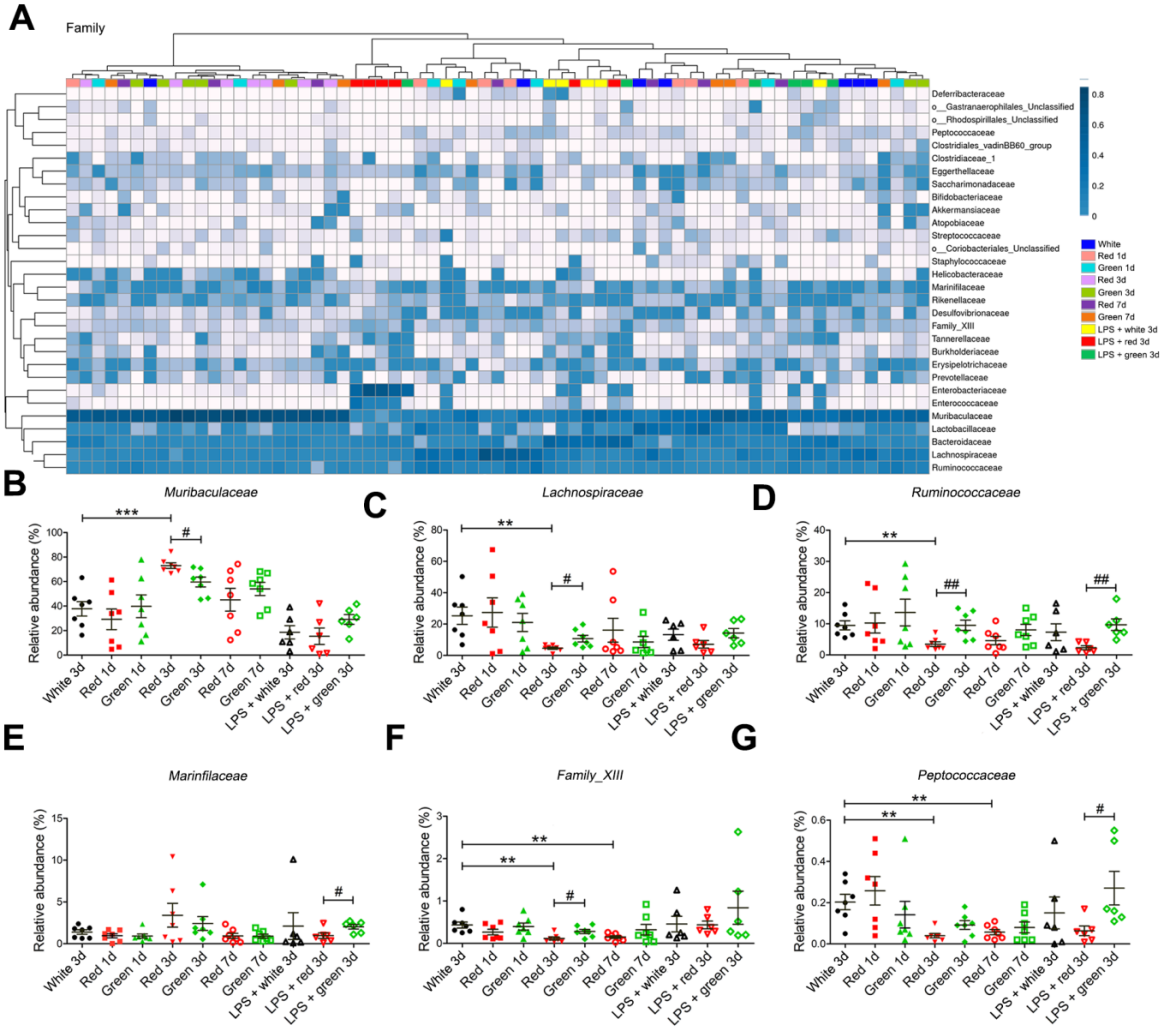
**Altered gut bacterial composition at the family levels.** (**A**) Relative abundance at the family level in the different groups. (**B**) Red light exposure increased the abundance of *Muribaculaceae* in non-LPS-treated mice on day 3 relative to ambient white light or green light exposure. (**C**) The abundance of *Lachnospiraceae* was lower in mice exposed to red light than in those exposed to ambient white light or green light. (**D**) Red light exposure reduced the abundance of *Ruminococcaceae* in non-septic mice on day 3 relative to ambient white light or green light exposure, as well as in septic mice on day 3 relative to green light exposure. (**E**) Red light exposure reduced the abundance of *Marinfilaceae* relative to green light exposure in septic mice on day 3. (**F**) Red light exposure reduced *Family-XIII* on days 3 and 7 relative to ambient white light exposure. LPS administration increased the abundance of *Family-XIII* in mice exposed to red light. (**G**) Red light exposure reduced the abundance of *Peptococcaceae* in non-septic mice on days 3 and 7 relative to ambient white light exposure, as well as in septic mice on day 3 relative to green light exposure. Data are presented as the mean ± SEM (n = 6-7). ^**^P < 0.01, ^***^P < 0.001, ^#^*P* < 0.05, ^##^*P* < 0.01.

At the family level, the red light-exposed group had a greater relative abundance of *Muribaculaceae* (Figure 5A, 5B; *P* < 0.001 and *P* < 0.05, respectively) but lower relative abundances of *Lachnospiraceae* (*P* < 0.01 and *P* < 0.05, respectively), *Ruminococcaceae* (both *P* < 0.01) and *Family-XIII* (*P* < 0.01 and *P* < 0.05, respectively) than the ambient white light- and green light-exposed groups on day 3 in non-septic mice (Figure 5C, 5D, 5F). The relative abundances of *Family-XIII* and *Peptococcaceae* in non-septic mice on days 3 and 7 were significantly lower in the red light-exposed group than in the ambient white light-exposed group (Figure 5F, 5G; all *P* < 0.01). In septic mice, the abundances of *Ruminococcaceae*, *Marinfilaceae* and *Peptococcaceae* were lower in the red light-exposed group than in the green light-exposed group on day 3 (Figure 5D, 5E and 5G; *P* < 0.01, *P* < 0.01 and *P* < 0.05, respectively).

**Figure 6:**
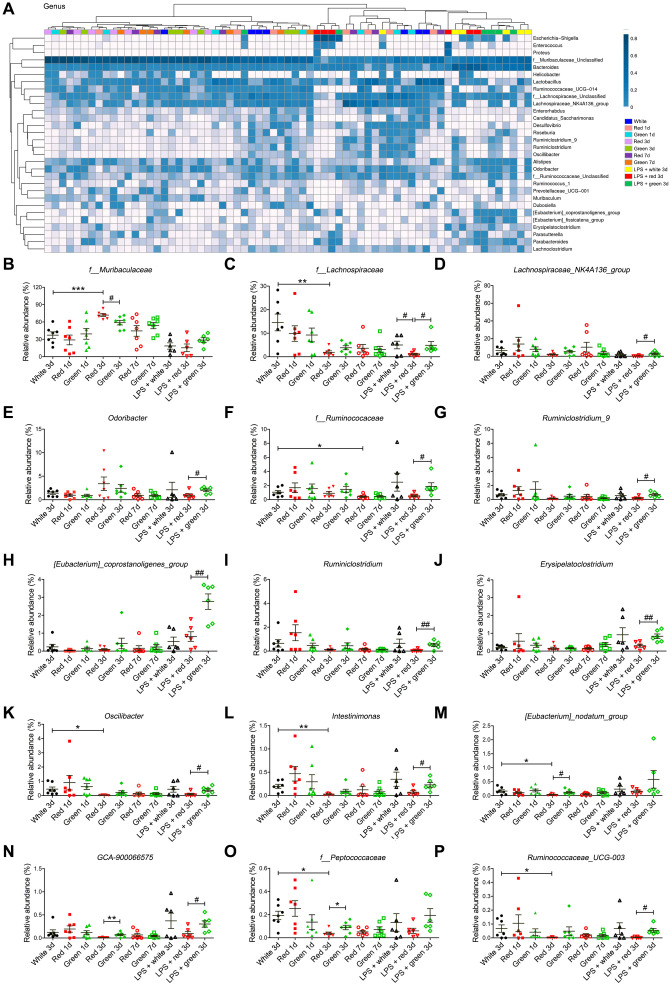
**Altered gut bacterial composition at the genus level.** (**A**) Relative abundance at the genus level in the different groups. (**B**) Red light exposure increased the abundance of *f_Muribaculaceae* in non-LPS-treated mice on day 3 relative to ambient white light or green light exposure. (**C**) Red light-exposed non-septic and septic mice had a lower abundance of *f_Lachnospiraceae* than ambient white light-exposed mice on day 3. (**D**) Red light exposure reduced the abundance of *Lachnospiraceae_NK4A136_group* in septic mice relative to green light exposure. (**E**) Red light exposure reduced the abundance of *Odoribacter* in septic mice relative to green light exposure. (**F**) Red light exposure reduced the abundance of *f_Ruminococcaceae* on day 3 relative to green light exposure in septic mice. Red light exposure reduced the abundances of *Ruminiclostridium_9* (**G**), *[Eubacterium]_coprostanoligenes_group* (**H**), *Ruminiclostridium* (**I**) and *Erysipelatoclostridium* (**J**) in septic mice relative to green light exposure. Red light exposure reduced the abundances of *Oscillibacter* (**K**) and *Intestinimonas* (**L**) in non-septic mice on day 3 relative to ambient white light exposure, as well as in septic mice on day 3 relative to green light exposure. (**M**) Red light exposure reduced the abundance of [*Eubacterium*]_*nodatum*_*group* in non-LPS-treated mice on day 3 relative to ambient white light or green light exposure. (**N**) Red light exposure reduced the abundance of *GCA-900066575* in both non-septic and septic mice on day 3 relative to green light exposure. (**O**) Red light exposure reduced the abundance of *f_Peptococcaceae* in non-septic mice on day 3 relative to ambient white light or green light exposure. (**P**) Red light exposure reduced the abundance of *Ruminococcaceae_UCG-003* in non-septic mice on day 3 relative to ambient white light exposure, as well as in septic mice on day 3 relative to green light exposure. Data are shown as the mean ± SEM (n = 6-7/group). ^*^P < 0.05, ^**^P < 0.01, ^***^P < 0.001, ^#^*P* < 0.05, ^##^*P* < 0.01, ^###^*P* < 0.001.

At the genus level, the abundances of *f_Muribaculaceae*, *f_Lachnospiraceae*, *f_Ruminococcaceae*, *Oscillibacter, Intestinimonas*, *[Eubacterium]_nodatum_group*, *f-Peptococcaceae* and *Ruminococcaceae-UCG-003* in non-septic mice on day 3 were remarkably lower in the red light-exposed group than in the ambient white light-exposed group (Figure 6A–6C, 6K–6M, 6O and 6P). In addition, the red light-exposed group had a higher abundance of *f_Muribaculaceae* and a lower abundance of *[Eubacterium]_nodatum_group*, *GCA-900066575* and *f-Peptococcaceae* than the green light-exposed group of non-septic mice on day 3 (Figure 6B, 6M–6O). In septic mice, the relative abundances of *f_Lachnospiraceae*, *Lachnospiraceae_NK4A136_group*, *Odoribacter*, *f-Ruminococcaceae*, *Ruminiclostridium-9*, *[Eubacterium]_* *coprostanoligenes_group*, *Ruminiclostridium*, *Erysipelato clostridium*, *Oscillibacter*, *Intestinimonas*, *GCA-900066575* and *Ruminococcaceae_UCG-003* were significantly lower in the red light-exposed group than in the green light-exposed group (Figure 6C–L, 6N and 6P). The relative abundance of *f_Lachnospiraceae* was significantly lower in the red light-exposed group than in the ambient white light-exposed group on day 3 in septic mice (Figure 6C; *P* < 0.05).

**Figure 7:**
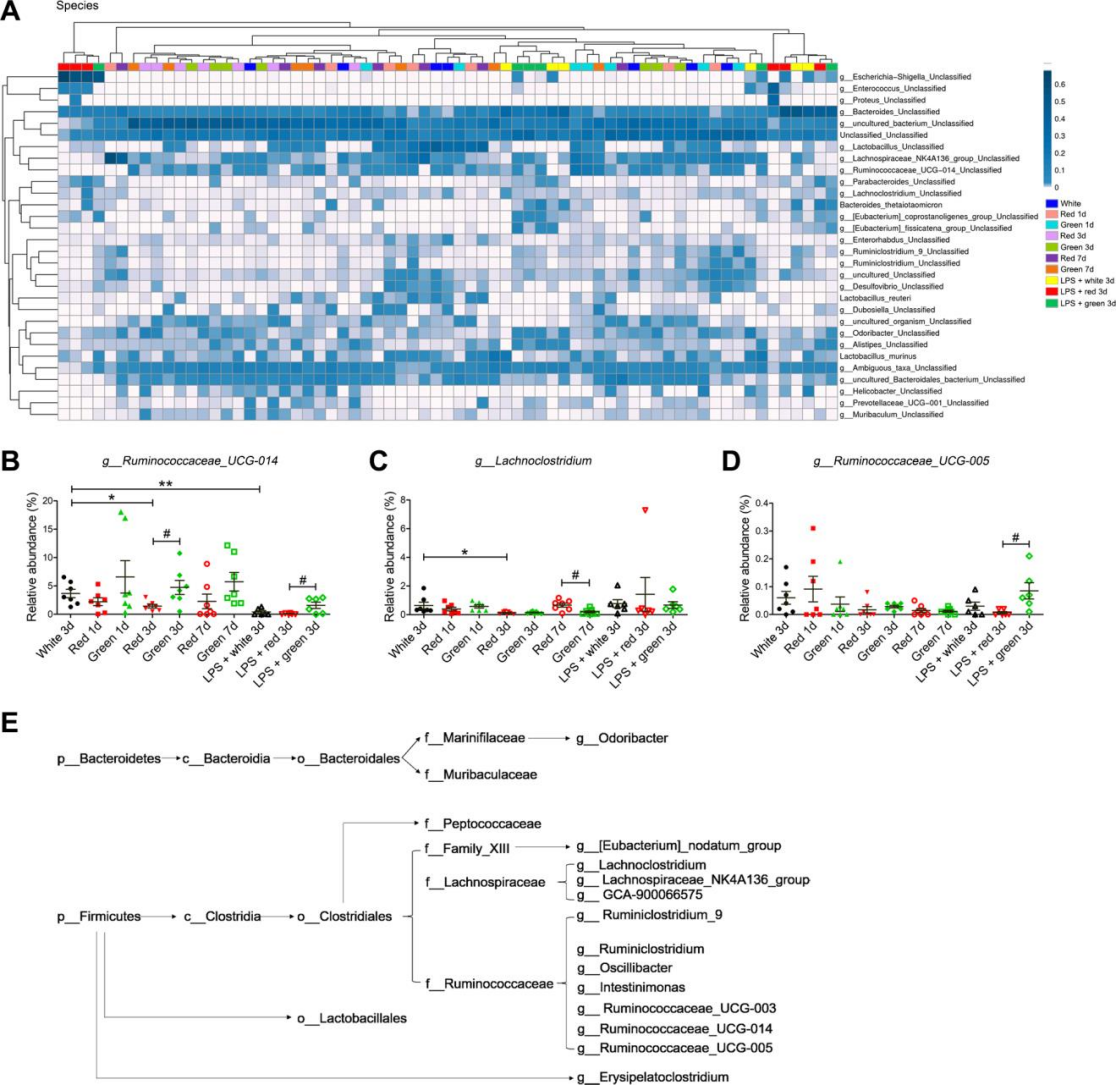
**Altered gut bacterial composition at the species level.** (**A**) Relative abundance at the species level in the different groups. (**B**) Red light exposure increased the abundance of *g_Ruminococcaceae_UCG-014* in non-LPS-treated mice on day 3 relative to ambient white light or green light exposure. (**C**) Red light exposure reduced the abundance of *g_Lachnoclostridium* in non-LPS-treated mice on day 3 relative to ambient white light exposure. (**D**) Red light exposure reduced the abundance of *g_Ruminococcaceae_UCG-005* in septic mice relative to green light exposure. (**E**) Phylogenetic *affiliations of the 31 altered bacteria.* Data are shown as the mean ± SEM (n = 6-7/group). ^*^P < 0.05, ^**^P < 0.01, ^#^*P* < 0.05, ^##^*P* < 0.01.

At the species level, the abundances of *g_Ruminococcaceae_UCG-014* and *g_Lachnoclostridium* in non-septic mice were significantly lower in the red light-exposed group than in the ambient white light-exposed group (Figure 7A–7C; both *P* < 0.05). In addition, the abundance of *g_Ruminococcaceae_UCG-014* in non-septic mice on day 3 was significantly lower in the red light-exposed group than in the green light-exposed group (Figure 7B; *P* < 0.05). In septic mice, the relative abundances of *g_Ruminococcaceae_UCG-014* and *g_Ruminococcaceae_UCG-005* were significantly lower in the red light-exposed group than in the green light-exposed group on day 3 (Figure 7B, 7D; both *P* < 0.05).

The above analyses demonstrated that the abundances of 31 bacteria at six phylogenetic levels were shifted to varying degrees in fecal samples from mice treated with LPS and exposed to different colors of light. The phylogenetic affiliations of these 31 bacteria are shown in Figure 7E. Our results demonstrated that red light exposure increased the abundance of phylum *Bacteroidetes* while reducing the abundance of phylum *Firmicutes*. However, LPS pre-administration reduced the abundance of some bacteria belonging to phylum *Bacteroidetes*, but increased the abundance of some bacteria belonging to phylum *Firmicutes*.

### Effects of the gut microbiota on red light-induced brain disorders after sepsis

**Figure 8:**
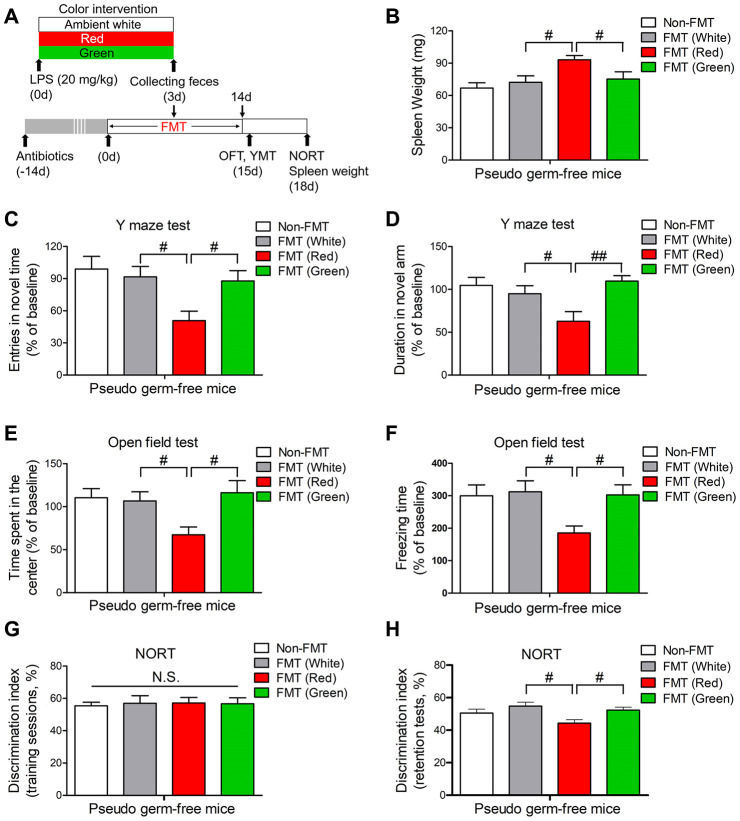
**Splenic and behavioral effects of fecal microbiota transplantation on pseudo germ-free mice.** (**A**) Treatment schedule. Mice received drinking water containing large doses of antibiotics for 14 consecutive days. Thereafter, the mice were orally treated with fecal microbiota from 20 mg/kg LPS-treated septic mice that had been exposed to light for three days. On days 15 and 18, behavioral tests were performed. Then, the mice were euthanized and their spleens were collected and weighed. (**B**) Pseudo germ-free mice transplanted with fecal microbiota from mice that had been treated with LPS and then exposed to red light displayed significant splenomegaly. They also exhibited remarkable cognitive impairment, as demonstrated by the reduced frequency of entering the novel arm (**C**) and the reduced time spent in the novel arm (**D**) in the Y maze test, the reduced time spent in the center (**E**) and the reduced freezing time (**F**) in the open field test, and the reduced time exploring the novel object (**G**, **H**) in the NORT. Data are shown as the mean ± SEM (n = 6-7/group). N.S., not significant, ^#^*P* < 0.05, ^##^*P* < 0.01.

In view of the striking alterations in the gut microbiota elicited by LPS and exposure to different colors of light, we then explored whether the gut microbiota influenced the development of cognitive dysfunction and anxiety-like behavior. We created a pseudo germ-free mouse model by administering large doses of antibiotics to mice for 14 consecutive days, and then we performed a fecal microbiota transplant (FMT) by gavaging the pseudo germ-free mice with the supernatants of fecal suspensions from septic mice exposed to different colors of light. Pseudo germ-free mice transplanted with fecal suspensions from red light-exposed septic mice had significantly heavier spleens than pseudo germ-free mice transplanted with fecal suspensions from ambient white light- or green light-exposed septic mice (Figure 8A, 8B; both *P* < 0.05). Furthermore, pseudo germ-free mice transplanted with fecal suspensions from red light-exposed septic mice exhibited impaired learning and increased anxiety compared with pseudo germ-free mice transplanted with fecal suspensions from ambient white light- or green light-exposed septic mice, as evidenced by their poorer performance in the open field test, Y maze test and NORT (Figure 8C–8H). These results confirmed that the alterations in the gut microbiota induced by red light exposure after sepsis impaired cognitive function and induced anxiety-like behavior.

### Gut dysbiosis induced by red light exposure stimulated brain disorders through the subdiaphragmatic vagus nerve

**Figure 9:**
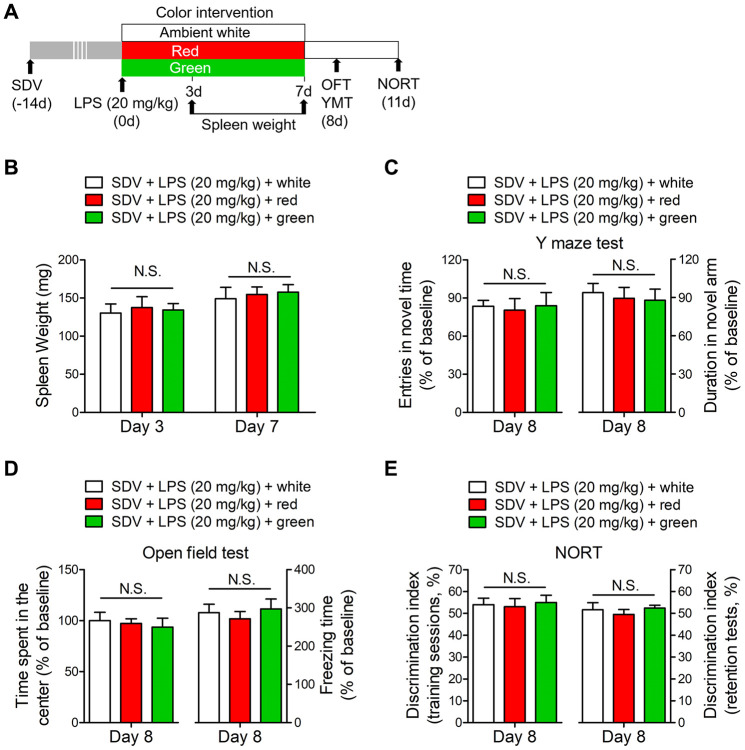
**SDV normalizes dysfunctional behaviors in red light-exposed septic mice.** (**A**) Treatment schedule. Mice underwent a splenectomy 14 days before LPS (20 mg/kg) administration. Thereafter, the mice were exposed to light for seven days. Some of the mice were euthanized and their spleens were collected and weighed on days 3 and 7. Open field and Y maze tests were conducted on day 8, and a NORT was performed on day 11. (**B**) Mice that underwent SDV displayed no significant differences in spleen weight following LPS treatment and light exposure. They also demonstrated no significant differences in behavioral performance, as evidenced by the results of the Y maze test (**C**), open field test (**D**) and NORT (**E**). Data are shown as the mean ± SEM (n = 6/group). N.S., not significant.

To further explore the underlying mechanisms of gut dysbiosis-induced brain disorders, we subjected mice to SDV 14 days prior to LPS administration. SDV abrogated the red light exposure-induced increases in spleen weight on days 3 and 7 after LPS administration (Figure 9A, 9B). Furthermore, SDV reversed the abnormal behaviors induced by red light exposure in the open field test, Y maze test and NORT (Figure 9C–9E). These data suggested that the subdiaphragmatic vagus nerve was crucial for red light exposure-induced brain disorders after sepsis.

**Figure 10:**
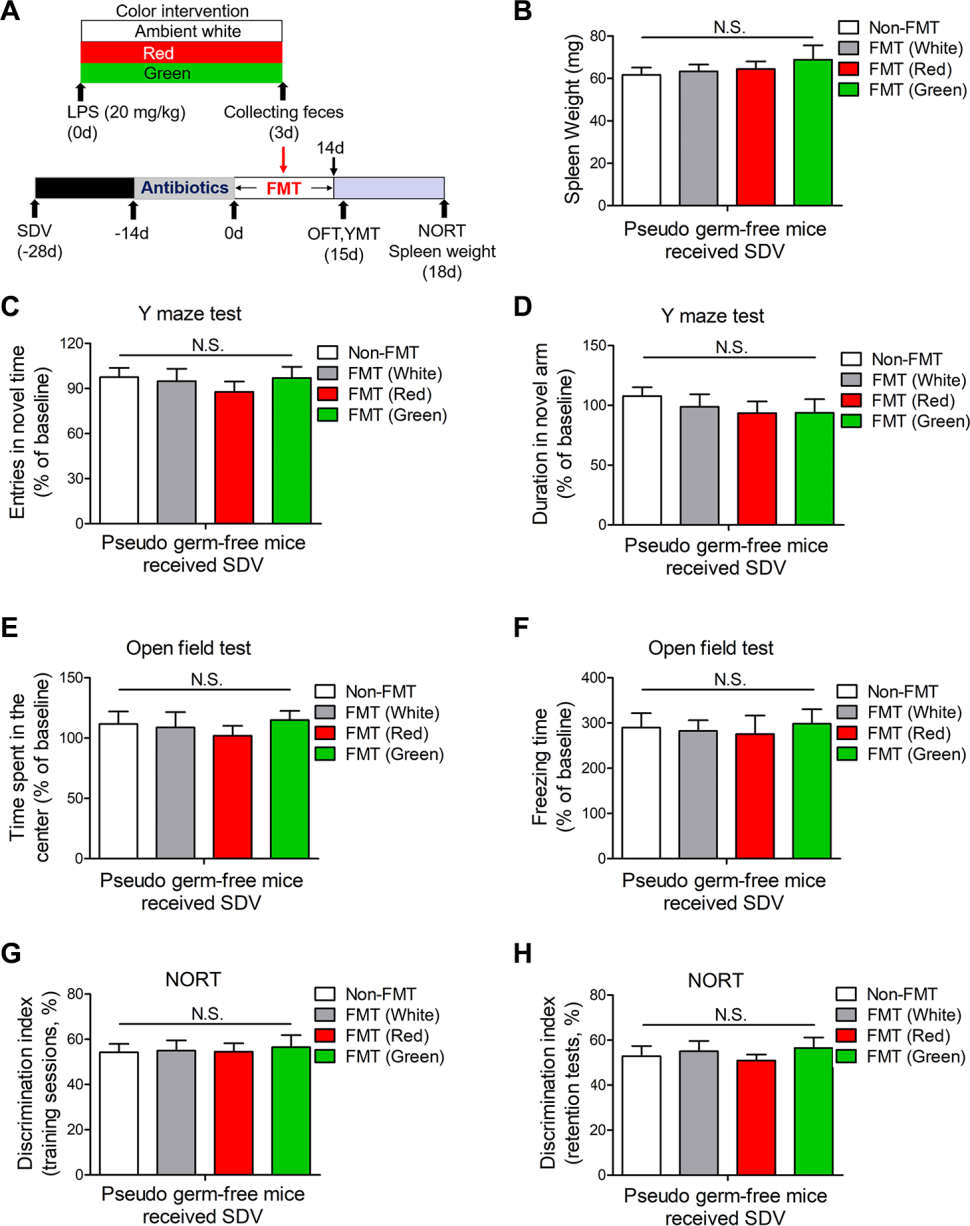
**Effects of SDV on pseudo germ-free mice transplanted with fecal microbiota.** (**A**) Treatment schedule. Mice underwent a splenectomy 28 days before FMT. After 14 days, the mice were given large doses of antibiotics for 14 days. Then, the mice were orally administered fecal microbiota from 20 mg/kg LPS-treated septic mice that had been exposed to light for three days. On days 15 and 18, behavioral tests were performed. Subsequently, the mice were euthanized, and their spleens were collected and weighed. (**B**) Pseudo germ-free mice that underwent SDV exhibited no significant differences in spleen weight after being transplanted with fecal microbiota from different groups. The mice also had similar behavioral performances, as evidenced by the lack of significant difference in the frequency of entering the novel arm (**C**) and the time spent in the novel arm (**D**) in the Y maze test, the time spent in the center (**E**) and the freezing time (**F**) in the open field test, and the time exploring the novel object (**G**, **H**) in the NORT. Data are shown as the mean ± SEM (n = 6/group). N.S., not significant.

We also found that splenomegaly did not occur in pseudo germ-free mice subjected to SDV and FMT with fecal suspensions from red light-exposed septic mice (Figure 10A, 10B). Moreover, SDV abrogated the abnormal behaviors in pseudo germ-free mice transplanted with fecal suspensions from red light-exposed septic mice (Figure 10C–10H). These results indicated that gut dysbiosis after sepsis contributed to red light exposure-induced cognitive dysfunction and anxiety-like behavior by signaling through the subdiaphragmatic vagus nerve.

## DISCUSSION

**Figure 11:**
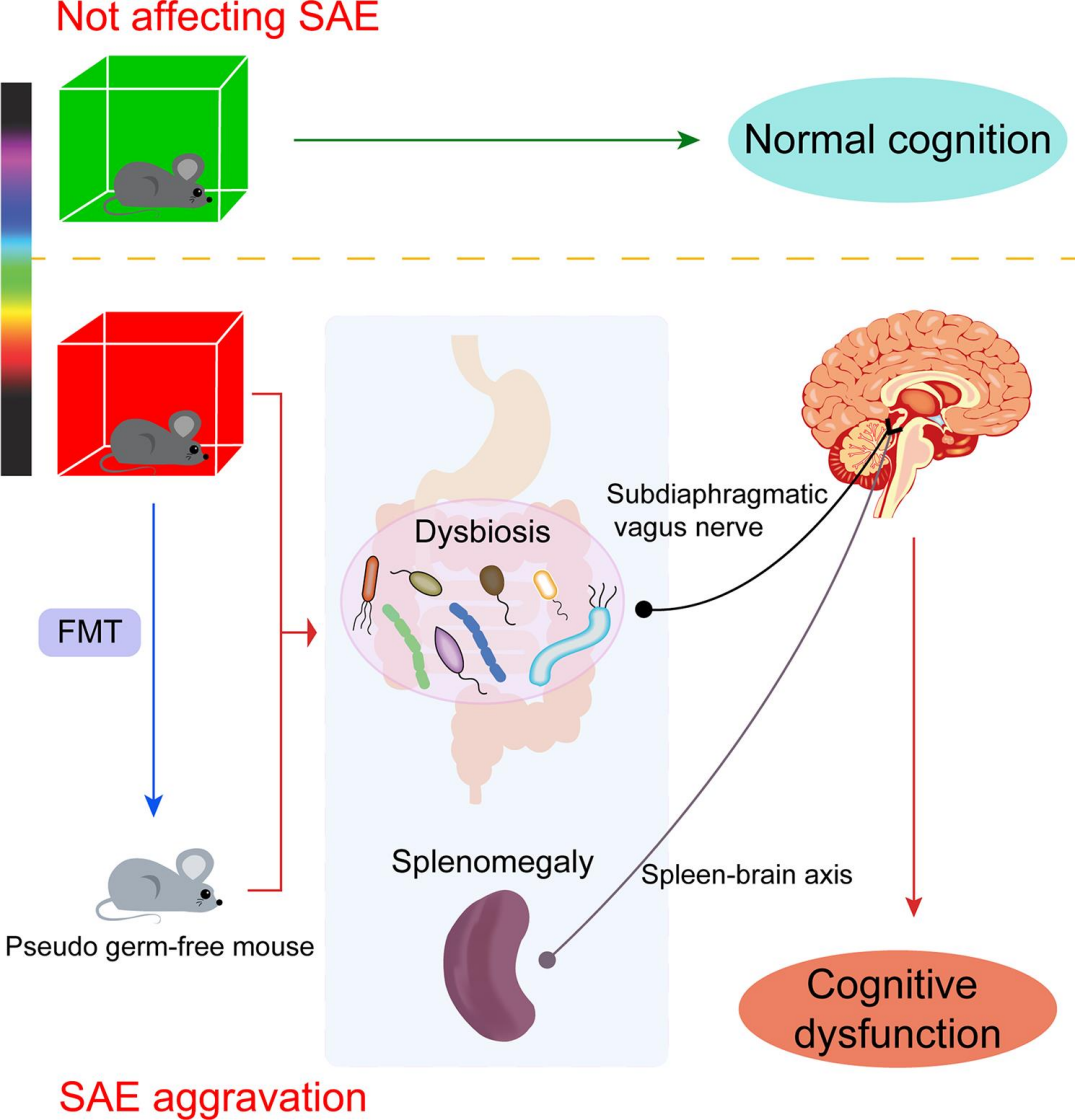
**The effects of light exposure on cognition are wavelength dependent.** Different colors of light distinctly influenced cognitive function in old mice. Red light exposure markedly altered the gut microbial diversity and composition and induced remarkable spleen enlargement in mice, leading to cognitive impairment in non-septic mice and exaggerating cognitive disorders in septic mice. Pseudo germ-free mice receiving fecal microbiota from red light-exposed septic mice also presented with splenomegaly and cognitive deficits. The damage depended on communication via the subdiaphragmatic vagus nerve through the “gut-brain axis” and the “spleen-brain axis.” Green light exposure had no obvious impact on cognition in mice.

In this study, we demonstrated that different colors of light had different effects on cognitive function. Red light exposure worsened spatial memory, learning and anxiety-like behavior after sepsis, while green light exposure had little impact on cognition and behavior after sepsis (Figure 11). A growing body of research has described the effects of the microbiome on host cognition and behavior [10]. The microbiome is an important contributor to emotional experiences, particularly anxiety and depression [31], as well as to cognitive processes such as learning and memory [10, 32, 33].

Given the established relationship between the microbiome and cognitive function, we analyzed the fecal microbial compositions of mice in different experimental groups using 16S ribosomal RNA (rRNA) gene sequencing. Our findings suggested that red light exposure after sepsis significantly altered the gut microbial composition in mice, whereas green or white light exposure after sepsis did not. The relative abundances of 31 bacteria differed significantly between the red light-exposed group and one or both of the other treatment groups. Among the bacteria that differed significantly, those of the orders *Lactobacillales, Clostridiales* and *Bacteroidales* and the family *Ruminococcaceae* have been reported to influence cognitive function [32, 34–36]. Furthermore, altered levels of bacteria from the phyla *Firmicutes* and *Bacteroidetes* have been associated with cognitive impairment [36, 37], in agreement with our findings. Thus, our data revealed that altered gut microbiota contributed to the red light-induced aggravation of cognitive dysfunction after sepsis.

To determine the causal link between the gut microbiome and cognitive impairment, we constructed a pseudo germ-free mouse model and transplanted the mice with fecal microbiota from mice exposed to different colors of light. As expected, pseudo germ-free mice transplanted with microbiota from red light-exposed septic mice exhibited the same abnormal behaviors as their donors, while those transplanted with microbiota from green light-exposed septic mice did not exhibit abnormal behaviors. These results suggested that the altered gut microbial composition induced by red light exposure after sepsis was the determinant of cognitive impairment.

To explore the communication pathway through which gut microbial dysbiosis induced brain disorders in mice, we performed SDV to determine the contribution of the vagus nerve. We found that SDV reversed the abnormal behaviors and splenomegaly elicited by red light-induced alterations of the intestinal flora. These data indicated that the subdiaphragmatic vagus nerve facilitates the communication between the microbiome and the brain. Our results were in line with previous studies demonstrating that the vagus nerve participates in the bidirectional communication between the gut microbiota and the brain in the so-called “gut-brain axis” [17–19].

In addition, it has been demonstrated that the cross-talk between the immune system and the brain contributes to the pathophysiology of many psychiatric conditions, including depression [38]. The spleen is the largest secondary immune organ in the body, and participates in the response to acute and chronic infections [39]. The “spleen-brain axis” is an important contributor to immune responses and behavior modulation [40, 41]. In a mouse model of chronic social defeat stress, susceptible mice had heavier spleens than control mice and chronic-social-defeat-stress-resilient mice, in agreement with our present results [42]. Importantly, the authors noted that the number of granulocytes in the spleen correlated positively with the weight of the spleen. We previously observed notable increases in spleen weight and proinflammatory cytokine levels in an LPS-induced depression-like phenotype model [43]. These two studies raise the possibility that spleen-derived inflammation promotes the development of brain disorders. In the present study, non-septic mice exhibited remarkable spleen enlargement following seven days of red light exposure, but not green light exposure, and septic mice exhibited splenomegaly after only three days of red light exposure. Notably, splenectomy abrogated the behavioral deficits induced by red light exposure after sepsis. These data indicated that the spleen is strongly associated with cognitive disorders.

The spleen serves as a reservoir of cytokine-producing monocytes [29, 30]. Splenomegaly in severe sepsis survivors was associated with the expansion of splenic leukocytes, among which Ly-6C^high^ monocytes strongly upregulated tumor necrosis factor in response to inflammatory stimuli [44]. Although there is controversy about whether splenectomy is protective or detrimental, numerous studies have demonstrated the protective effects of splenectomy in a variety of murine disease models, including models of stroke, sepsis and repeated social defeat [29, 45, 46]. For example, in a mouse model of repeated social defeat, splenectomy blocked monocyte trafficking from the spleen to the brain and prevented recurring anxiety-like behavior [29]. We hypothesize that the gut microbial alterations induced by red light exposure after sepsis may have triggered inappropriate immune responses in the spleen, which subsequently promoted the spread of peripheral inflammation to the brain, ultimately causing cognitive impairment. However, the details of the potential mechanisms need to be investigated further.

It is now commonly recognized that melanopsin-expressing ipRGCs dominantly participate in non-visual responses to light exposure. The extensive and interactive efferent projections of ipRGCs involve subcortical areas (the hypothalamus, brainstem and thalamus) and limbic structures (the amygdala and hippocampus), which have been implicated in cognitive performance [29]. Human studies have demonstrated that brain activities associated with cognitive tasks are altered by light exposure in a wavelength-dependent manner, being more sensitive to blue light than to violet, red or green light [27, 47]. Pilorz et al. suggested that different wavelengths of light distinctly altered sleep-wake patterns, with blue light leading to alertness and green light rapidly inducing sleep. In a mouse model of sepsis, blue light exposure for 24 hours enhanced bacterial clearance and reduced systemic inflammation, whereas red light exposure for 24 hours had no significant effects on these measures [48]. However, in our study, continuous red light exposure for seven days aggravated cognitive dysfunction after sepsis, while green light exposure did not. Thus, our results appear to conflict with previous reports that melanopsin-expressing ipRGCs are minimally sensitive to red light. However, at an intensity above 20 lux, red light has been reported to enhance sleep induction [49]; thus, red light may be a key contributor to certain pathophysiological responses.

There were limitations to our present study. We only assessed the effects of two narrow-band monochromatic wavelengths of light (red and green) on cognitive function. The effects of shorter wavelengths of light on cognitive function should be further explored. Nevertheless, our study has revealed the unique effects of red light on the brain – namely, that prolonged red light exposure can potently impair cognitive function, especially in patients with SAE. Post-septic exposure to green light had no significant effects on the diversity and composition of the gut microbiota or on the weight of the spleen, which may explain its non-significant effects on cognitive function and anxiety-like behavior. Our findings highlight the importance of avoiding red light exposure in daily life for the sake of cognitive function, and provide evidence that an altered gut microbiome is an essential contributor to SAE.

## MATERIALS AND METHODS

### Animals

Old male C57BL/6J mice (78 weeks old) were purchased from Vital River Laboratory Animal Technology Co Ltd., Beijing, China. The mice were housed under specific pathogen-free conditions, maintained on a 12-hour light/dark cycle (lights on 6:00 AM-6:00 PM), and provided with food and water *ad libitum*. All procedures were performed in accordance with the U.K. Animals (Scientific Procedures) Act, 1986. All animal protocols were approved by the Committee of Experimental Animals of Tongji Medical College (Wuhan, China).

### Experimental grouping and treatment

The experiment was performed in five parts. In part A, mice were intraperitoneally injected with LPS (10 or 20 mg/kg; L-4130, serotype 0111:B4; Sigma-Aldrich, St Louis, MO, USA) or 0.9% saline (10 or 20 mL/kg) before being exposed to red, green or ambient white light. The mice that were exposed to red or green light for one, three or seven days were housed in transparent cages with red- or green-painted walls, allowing red or green light to pass through (Figure 1A, 1B). Some mice were euthanized on day 1, 3 or 7 of their exposure to different colors of light so that their spleens could be collected. Others were subjected to the Y maze and open field tests on day 8 and the NORT on day 11 after the commencement of light exposure. Stool samples were collected in sterile microtubes, frozen immediately in liquid nitrogen and stored at −80° C for 16S rRNA analysis or FMT.

In part B, mice underwent a splenectomy or a sham operation 14 days before LPS (20 mg/kg) administration, and then were exposed to red, green or ambient white light for seven days after LPS treatment. Y maze and open field tests were conducted on day 8, and a NORT was performed on day 11 after the commencement of light exposure.

In part C, we first prepared pseudo germ-free mice as recipients for the FMT experiments, as follows. Broad-spectrum antibiotics (ampicillin 1 g/L, neomycin sulfate 1 g/L and metronidazole 1 g/L; Sigma-Aldrich) dissolved in drinking water were given *ad libitum* to C57BL/6 mice for 14 consecutive days. The drinking solution was renewed every two days. After their commensal gut microflora had been depleted, the mice were given fecal microbiota from LPS (20 mg/kg)-treated mice exposed to 3 days of red, green or ambient white light for 14 consecutive days. Y maze and open field tests were conducted on day 15, and a NORT was performed on day 18 after the commencement of the FMT. Subsequently, the mice were euthanized, and their spleens were collected and weighed.

In part D, mice were subjected to a total SDV 14 days before LPS administration. They were then administered 20 mg/kg LPS and exposed to different colors of light for three or seven days. On days 3 and 7 after the commencement of light exposure, the spleens of some mice were collected and weighed. In other mice, Y maze and open field tests were conducted on day 8 and a NORT was performed on day 11 after the commencement of light exposure.

In part E, mice were subjected to SDV before being prepared for pseudo germ-free modeling. After a 14-day recovery period, antibiotics were administered for 14 days and FMT experiments were performed as described above. On day 15 after the commencement of the FMT, Y maze and open field tests were conducted. The NORT was performed on day 18. Then the mice were euthanized, and their spleens were collected and weighed.

### Behavior tests

### Open field test

Open field tests were used to assess exploratory activity, non-associated memory and anxiety-like behavior. A video camera was placed directly above the open field chamber (40 × 40 × 40 cm). The mouse was gently placed in the center of the chamber under dim light and allowed to move freely for five minutes. The time (seconds) spent in the center of the field (middle 20 × 20 cm area) and the freezing time (seconds) were analyzed. The chamber floor was cleaned with 70% ethanol between each test.

### Y maze task

Short-term spatial working memory was assessed using a gray plastic Y maze apparatus consisting of three identical open arms (width 8 cm × length 30 cm × height 15 cm) at an angle of 120°. The three arms included a starting arm, second arm and novel arm (blocked during the first trial, open during the second trial). Different visual cues (circles, triangles and pentagrams) were placed at the end of each arm and were constant throughout the trials. A video camera was placed directly above the Y maze apparatus.

The Y maze test consisted of two trials separated by a two-hour interval. In the first (training) trial, the mouse was placed in the starting arm and allowed to explore it and the second arm for 10 minutes. In the second trial, the mouse was placed in the starting arm and allowed to explore all three arms freely for five minutes. An arm entry was defined as all four paws of the mouse being in the arm. The number of entrances into the novel arm and the duration spent in the novel arm were analyzed. Each arm was cleaned with 70% ethanol between each trial.

### NORT

A NORT was performed to assess recognition memory. The mouse was placed in a gray plastic chamber (35 x 25 x 35 cm) for 30 minutes two days before the NORT. During the training sessions, two identical objects were placed symmetrically in the center of the chamber, and the mouse was allowed to explore for 10 minutes. The time spent exploring each object was recorded. During the retention tests, one of the familiar objects was replaced by a novel object, and the mouse was allowed to explore the chamber freely for five minutes. The mouse was considered to be exploring an object when it faced the object at a distance of ≤ 1 cm. The discrimination index was determined as the time spent exploring any one object (training session) or the novel object (retention session) divided by the total time spent exploring both objects.

### Splenectomy

Total splenectomies were performed under isoflurane anesthesia. A 2-cm subcostal incision was made on the left dorsolateral side of the abdomen, and the spleen was carefully exteriorized through the incision. The afferent and efferent vessels near the spleen were ligated using 6-0 silk sutures, and then the spleen was removed. The abdominal wall was closed with 4-0 silk sutures, and the skin was stitched using 3-0 silk sutures. Sham-splenectomized mice underwent the same procedures without the removal of the spleen.

### Total SDV

Vagotomies were performed as previously described [50]. Briefly, a midline abdominal incision was made and the esophagus was exposed, while the costal arc, liver and stomach were carefully kept out of sight. Then, under a surgical microscope, the dorsal and ventral branches of the vagus were dissected along the subdiaphragmatic esophagus. Each vagal branch was ligated twice with surgical thread at an interval of 1-2 cm and then dissected between the ligatures. The incision was then closed with running sutures along the abdominal wall and stop sutures along the skin. Fourteen days after the operation, the observation of an increased stomach size indicated a successful SDV. For sham surgery, the trunk of the vagus nerve was gently exposed but not cut.

### Fecal microbiota transplant

The mice were placed in a clean cage with sterilized filter paper on the bottom. Stool samples from the experimental mice were collected in a sterile microtube immediately after defecation and promptly stored at −80° C until analysis. The filter paper was changed for different mouse samples. For the preparation of fecal microbiota, 1 g of feces from a donor mouse was diluted in 10 mL of sterile phosphate-buffered saline. A suspension was made from this mixture, and each recipient mouse was given 0.2 mL by gavage.

### 16S rRNA analysis

Fecal samples were collected, placed in 1.5-mL tubes, snap-frozen on dry ice and stored at −80° C. The 16S rRNA analysis of the fecal samples was performed by GENEWIZ Biotech Co., Ltd. (Suzhou, China). Briefly, total genomic DNA was extracted from the samples using a Soil DNA kit, and then was amplified in 25-μL triplicate reactions with bacterial 16S rRNA gene (V3-V4 region)-specific forward primers containing the sequence “CCTACGGRRBGCASCAGKVRVGAAT” and reverse primers containing the sequence “GGACTACNVGGGTWTCTAATCC”. The amplified DNA libraries were validated and quantified for sequencing on an Illumina MiSeq instrument (Illumina, San Diego, CA, USA). Through quality filtering out, the effective sequences were grouped into operational taxonomic units. Alpha diversity indexes were calculated from rarefied samples on the Quantitative Insights Into Microbial Ecology (QIIME) website. The Shannon index was used for diversity, while the Chao 1 index was used for richness. Beta diversity was calculated using weighted and unweighted UniFrac, and principal coordinate analysis performed. An Unweighted Pair Group Method with Arithmetic mean tree was built from the beta diversity distance matrix.

### Statistical analysis

Data are expressed as the mean ± standard error of the mean (SEM). For group comparisons, statistical significance was tested with one-way analysis of variance followed by Tukey’s multiple comparison post-hoc tests. In Figure 2F–2K, data were analyzed using two-way analysis of variance with Tukey’s post-hoc analysis. Survival was analyzed with the Mantel-Cox log-rank test. *P* < 0.05 was considered statistically significant. Statistical analyses were performed using GraphPad Prism 8 software.
