# Correction for: Red light exaggerated sepsis-induced learning impairments and anxiety-like behaviors

**DOI:** 10.18632/aging.204240

**Published:** 2022-08-14

**Authors:** Bing Xie, Yujing Zhang, Hong Qi, Hua Yao, You Shang, Shiying Yuan, Jiancheng Zhang

**Affiliations:** 1 Department of Critical Care Medicine, Union Hospital, Tongji Medical College, Huazhong University of Science and Technology, Wuhan, 430022, China; 2 Institute of Anesthesia and Critical Care Medicine, Union Hospital, Tongji Medical College, Huazhong University of Science and Technology, Wuhan, 430022, China

**This article has been corrected:** The authors noticed an error in **Figure 1**. As a result of misfiling the data, the graph in panel **1F**, which should show the reduced time spent in the center in the open field test, was the same as the graph in panel **1G**, which shows the reduced freezing time in that test. The authors corrected panel **1F** in **Figure 1** by using the correct graph from the original sets of experiments. The authors stated that this alteration does not affect the results or conclusions of this work and apologized for any inconvenience caused.

New **Figure 1** is presented below.

**Figure 1:**
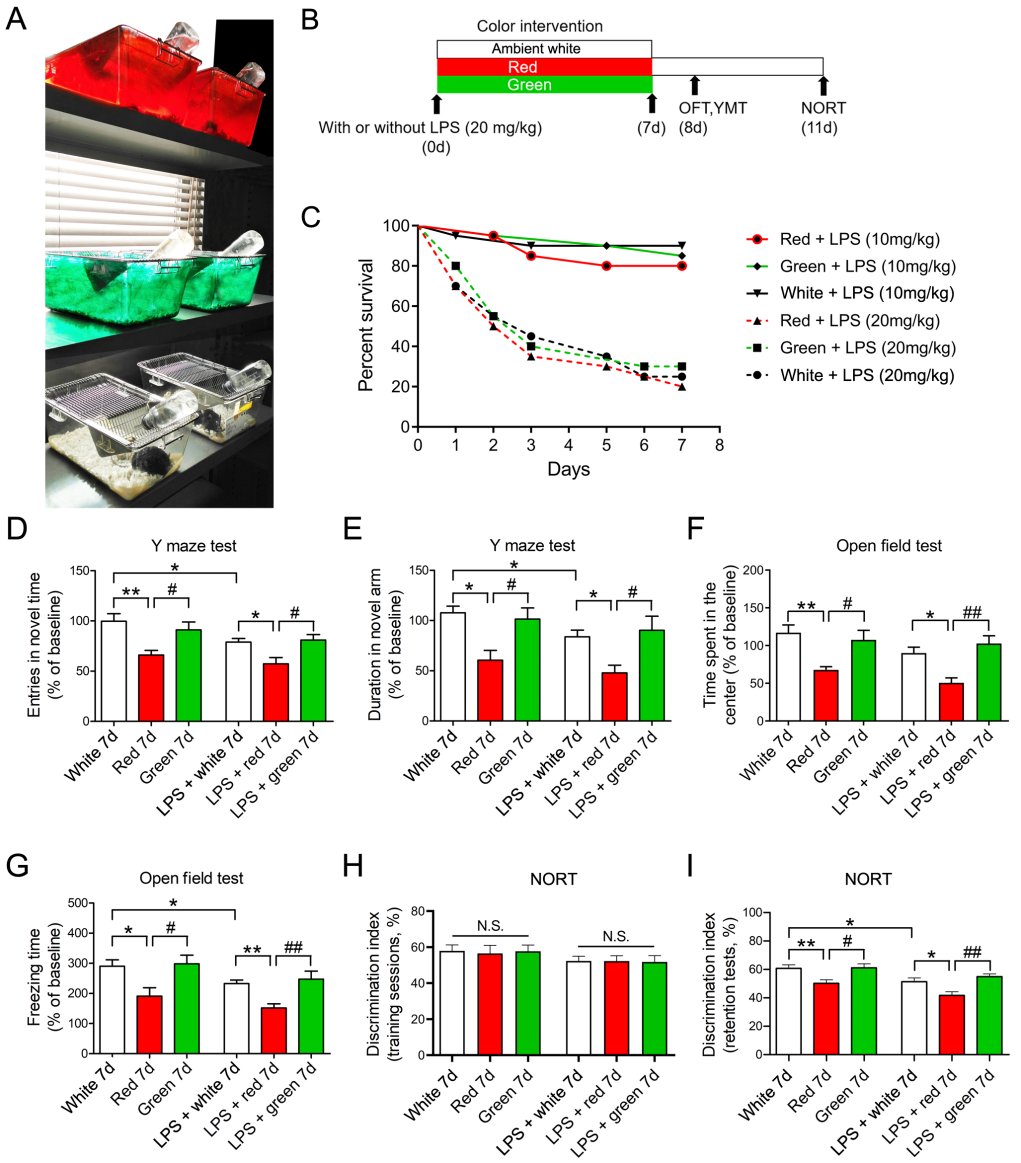
**Red light exposure induces cognitive impairment in both non-LPS-treated and LPS-treated mice.** (**A**, **B**) Treatment schedule. Mice were treated with or without LPS (20 mg/kg) and then exposed to light for 7 days. Non-LPS-treated mice and LPS-treated mice were scheduled for open field and Y maze tests on day 8 and a NORT on day 11. (**C**) Survival curves (n = 20/group). The survival rate did not significantly differ among groups exposed to red, green or ambient white light for 7 days after administration of LPS (10 or 20 mg/kg). (**D**–**I**) Red light exposure impaired spatial learning and memory and induced anxiety-like behavior as compared to ambient white or green light. This is evidenced by the reduced frequency of entering the novel arm (**D**) and the reduced time spent in the novel arm (**E**) in the Y maze test, the reduced time spent in the center (**F**) and the reduced freezing time (**G**) in the open field test, and the reduced time exploring the novel object (**H**, **I**) in the NORT. Data are shown as the mean ± SEM (n = 6-8/group). *P < 0.05, **P < 0.01, ^#^*P* < 0.05, ^##^*P* < 0.01.

